# A Conversation With ChatGPT About the Usage of Lithium in Pregnancy for Bipolar Disorder

**DOI:** 10.7759/cureus.46548

**Published:** 2023-10-05

**Authors:** Jaismeen Randhawa, Aadil Khan

**Affiliations:** 1 Psychiatry, Sri Guru Ram Das Institute of Medical Sciences and Research, Amritsar, IND; 2 Trauma Surgery, OSF Saint Francis Medical Center, University of Illinois Chicago, Peoria, USA; 3 Cardiology, University of Illinois Chicago, Illinois, USA; 4 Internal Medicine, Lala Lajpat Rai Hospital, Kanpur, IND

**Keywords:** bipolar disorder, pregnancy, lithium, chat generative pre-trained transformer (chatgpt), artificial intelligence (ai)

## Abstract

This conversation with ChatGPT explores the use of lithium in pregnancy for bipolar disorder, a topic of significant importance in psychiatry. Bipolar disorder is characterized by extreme mood swings, and its prevalence varies globally. ChatGPT provides valuable information on bipolar disorder, its prevalence, age of onset, and gender differences. It also discusses the use of lithium during pregnancy, emphasizing the need for individualized decisions, close monitoring, and potential risks and benefits. However, it is essential to note that ChatGPT’s responses lack specific references, raising concerns about the reliability of the information provided. Further research is needed to quantify the correctness and dependability of ChatGPT-generated answers in the healthcare context.

## Editorial

OpenAI is an artificial intelligence (AI) research and deployment company based in San Francisco. Their goal is to build software and artificial general intelligence benefiting humanity. ChatGPT is an app built by OpenAI. ChatGPT is a language-based chatbot, which stands for Chat Generative Pre-Trained Transformer (ChatGPT), launched on November 30, 2022. It is one of the most advanced and powerful language models as of the last update in September 2021. ChatGPT’s popularity can be attributed to its versatility, intelligence, and ability to engage in human-like conversations [1]. Since its November 2022 debut, ChatGPT has provoked substantial conversations in the healthcare industry. Psychiatry has benefited from deep learning, and the GPT language model is a potent deep learning-based language model with enormous potential for this subject [2].

Bipolar disorder is a brain disorder that causes changes in a person’s mood, energy, and ability to function. People with bipolar disorder experience intense emotional states that typically occur during distinct periods of days to weeks, called mood episodes. These mood episodes are categorized as manic/hypomanic (abnormally happy or irritable mood) or depressive (sad mood) or can have periods of neutral mood as well. When treated, people with bipolar disorder can lead full and productive lives.

Most mood-stabilizing drug plasma concentrations drop during pregnancy due to physiological changes [3]. The likelihood of prolonged symptom remission throughout pregnancy is increased by routine symptom and medication concentration monitoring for lithium and anticonvulsants with reflexive dose modification. The frequency of laboratory monitoring and dose modification for psychiatric medications is determined by the elimination clearance trajectory throughout pregnancy. Lithium, lamotrigine, carbamazepine, and atypical antipsychotic pharmacokinetics during pregnancy and postpartum.

According to our observations, ChatGPT generates thorough writing with generally accurate information in a simple manner. It is intriguing to note that ChatGPT presents accurate facts. It also offers details on prescription drugs.

Bipolar patient worries are addressed with ChatGPT, which offers sympathetic responses. The lack of acknowledged sources or references to confirm the material in these comments, however, creates a challenge to their legitimacy.

Although ChatGPT has a significant future in clinical information retrieval, it is constrained by the fact that the responses it produces are not settled (Table 1). It can be used as a tool for supportive and empathetic measures. The correctness and dependability of ChatGPT-generated answers to patient queries should be quantified in future studies.

**Table 1:** Conversation with Chat Generative Pre-Trained Transformer (ChatGPT) about the usage of lithium in pregnancy for bipolar disorder.

Questions	Response by ChatGPT
1. What is bipolar disorder?	Bipolar disorder, also known as manic-depressive illness, is a mental health condition characterized by extreme shifts in mood, energy levels, and activity levels. These shifts include episodes of depression and episodes of mania or hypomania.
2. What is the prevalence of bipolar?	The prevalence of bipolar disorder varies across different populations and geographical regions. Globally, the estimated lifetime prevalence of bipolar disorder is around 1-2%, which means that 1 to 2 out of every 100 individuals may experience bipolar disorder at some point in their lives.
3. What is the age of onset of bipolar?	Bipolar disorder typically emerges in late adolescence to early adulthood, although it can start at any age. The average age of onset is often in the late teens to early twenties. However, some individuals may experience their first episode of bipolar disorder during childhood or later in life.
4. What is the gender difference?	Bipolar disorder appears to affect both genders, but there are some gender-related differences: Bipolar I Disorder is equally common in both males and females. Bipolar II Disorder might be more prevalent among females. Some studies suggest that males might have an earlier onset of the disorder compared to females.
5. What are the comorbidities?	Bipolar disorder often occurs alongside other mental health conditions, including anxiety disorders, substance use disorders, and attention-deficit/hyperactivity disorder (ADHD). The presence of comorbid conditions can complicate diagnosis and treatment.
6. What is the dosage of lithium?	The dosage of lithium can vary widely depending on the individual's needs and response to the medication. During pregnancy, the goal is often to use the lowest effective dose to manage bipolar symptoms while minimizing potential risks. In some cases, a healthcare provider might consider reducing the dosage or even discontinuing lithium during pregnancy, depending on the severity of the disorder and the potential risks involved.

7. What are the effects of lithium in pregnancy?	
Risks	
Risk category	Details
Congenital malformations	- There is some evidence to suggest that lithium use during the first trimester of pregnancy might be associated with a slightly increased risk of certain congenital malformations, particularly heart defects.
Neonatal complications	- Babies born to mothers who have taken lithium during pregnancy might be at risk of experiencing neonatal complications, including neonatal lithium toxicity, which can affect their heart and thyroid function.
Benefits	
Benefit category	Details
Managing bipolar symptoms	- Bipolar disorder can pose significant risks to the pregnant person and the developing fetus if left untreated. Mood episodes, stress, and unmanaged mental health conditions can negatively impact prenatal care and overall well-being.
Stabilizing mood	- Lithium is a well-established mood stabilizer that can help manage manic and depressive episodes associated with bipolar disorder.
Considerations	
Consideration category	Details
Individualized decision	- The decision to use lithium during pregnancy should be based on an individual assessment of the pregnant person's medical history, the severity of their bipolar disorder, and the potential risks and benefits of treatment.
Alternative medications	- In some cases, healthcare providers might consider alternative medications with a more established safety profile during pregnancy, such as certain antipsychotic medications or other mood stabilizers.
Management	
Management category	Details
Close monitoring	- If a pregnant person and their healthcare provider decide that lithium is the best option, close monitoring of lithium levels and overall health throughout pregnancy is essential to minimize risks.
Adjustments	- Dosage adjustments might be necessary during pregnancy due to changes in the body's metabolism. Medications like mood stabilizers, antipsychotics, and antidepressants are commonly used.
Psychotherapy	- Psychotherapy, particularly cognitive-behavioral therapy (CBT) and psychoeducation, can be effective in developing coping strategies.
